# In Vitro Antibacterial Efficacy of Recombinant Phage-Derived Endolysin LysTAC1 Against Carbapenem-Resistant *Acinetobacter baumannii*

**DOI:** 10.3390/antibiotics14100975

**Published:** 2025-09-26

**Authors:** Inam Ullah, Song Cui, Qiulong Yan, Hayan Ullah, Shanshan Sha, Yufang Ma

**Affiliations:** 1 Department of Biochemistry and Molecular Biology, Dalian Medical University, Dalian 116044, China; inammicro09@gmail.com (I.U.); hayan.khan12@gmail.com (H.U.); shanshan_sha@dmu.edu.cn (S.S.); 2 Department of Critical Care Medicine, Dalian Municipal Central Hospital, Central Hospital of Dalian University of Technology, Dalian 116021, China; cuisong20082025@163.com; 3 Department of Microbiology, Dalian Medical University, Dalian 116044, China; qiulongyan@dmu.edu.cn

**Keywords:** endolysin, expression, antibiotic resistance, *Acinetobacter baumannii*, in silico analysis

## Abstract

**Background:** The rapid emergence of antibiotic resistance in *Acinetobacter baumannii* has led the World Health Organization (WHO) to designate it as a “high priority” pathogen. The emergence of multidrug-resistant (MDR) and pandrug-resistant (PDR) strains poses considerable treatment challenges. As antimicrobial resistance (AMR) escalates toward a post-antibiotic era, innovative therapeutic solutions are urgently needed. **Objectives:** To clone, over-express, and characterize a novel endolysin, LysTAC1, from *Acinetobacter* phage TAC1 for its antibacterial efficacy against multidrug-resistant bacteria. **Methods:** A 24 kDa endolysin featuring a glycoside hydrolase Family 19 chitinase domain was tested against carbapenem-resistant *Acinetobacter baumannii* clinical isolates and various *Escherichia coli* strains following outer membrane permeabilization with Ethylenediaminetetraacetic acid (EDTA). Stability assays and molecular docking studies were performed. **Results:** LysTAC1 demonstrated potent lytic activity against Gram-negative bacteria but showed no activity against Gram-positive bacteria (*Staphylococcus aureus* ATCC 29213 and *Enterococcus gallinarum* HCD 28-1). LysTAC1 maintained activity across pH 6–9 and temperatures 4–65 °C, with differential sensitivity to metal ions where K^+^ showed no inhibitory effect at any concentration (0.1–100 mM), and Fe^2+^ was non-inhibitory at lower concentrations (0.1–1 mM), while Mg^2+^ and Ca^2+^ demonstrated concentration-dependent inhibition across the tested range (0.1–100 mM). Molecular docking revealed LysTAC1 interactions with chitinase substrates 4-nitrophenyl N-acetyl-β-D-glucosaminide and 4-nitrophenyl N, N-Diacetyl-β-D-chitobioside, with binding energies of −5.82 and −6.85 kcal/mol, respectively. **Conclusions**: LysTAC1 shows significant potential as a targeted therapeutic agent against *A. baumannii* with robust stability under physiological conditions.

## 1. Introduction

The genus *Acinetobacter* includes over 60 validly recognized species and has been classified in the family *Moraxellaceae*, order *Pseudomonadales*, and class *Gammaproteobacteria* since 1991 [1,2]. Smith in 2007 initially sequenced the complete genome of *A. baumannii* (ATCC 17978) [3]. However, routine diagnostic laboratories have faced challenges in accurately identifying *A. baumannii* due to its phenotypic similarities and close phylogenetic relationship with several other species of *Acinetobacter* [1]. The *Acinetobacter calcoaceticus–A. baumannii* (Acb) complex comprises six recognized species: *A. calcoaceticus* (formerly *Acinetobacter* genomic species 1), *A. baumannii*, *A. pittii* (previously *Acinetobacter* genomic species 3), *A. nosocomialis* (previously *Acinetobacter* genomic species 13TU) [4], *A. seifertii* (previously *Acinetobacter* genomic species “close to 13TU”) [5], and presently recognized *A. lactucae* (previously *Acinetobacter* NB14, also known as *A. dijkshoorniae*) [1,6,7,8]. Among these, *A. calcoaceticus* is non-pathogenic, whereas the other five species are clinically relevant and are commonly referred to as *A. baumannii* or the *A. baumannii* group in clinical microbiology. The prevalence of specific species within the *A. baumannii* group varies by geography [9,10], although *A. baumannii* is considered the most virulent from a clinical standpoint [11,12]. *A*. *baumannii* is a Gram-negative, non-motile, non-fermenting, and oxidase-negative coccobacillus often found in habitats like sewage, water, and healthcare settings [13]. This pathogen is recognized for causing multiple healthcare-associated infections, which include bacteremia, pneumonia, urinary tract infections, and skin or soft tissue infections [14]. The growing concern of *A. baumannii* arose significantly after outbreaks among U.S. military personnel during the Iraq conflicts and has subsequently evolved into a global issue [15]. The rapid emergence of antibiotic resistance in *A. baumannii* has led the World Health Organization (WHO) to designate *A. baumannii* as a “high priority” pathogen. In recent decades, multidrug-resistant (MDR) and pandrug-resistant (PDR) strains of *A. baumannii* have emerged, posing considerable challenges in treatment [14,16]. Since the 1990s, the prevalence of *A. baumannii* in hospital environments has been consistently recorded, particularly in areas with a high incidence of carbapenem resistance [17]. 

Bacteriophages are considered an effective biological agent for the eradication of, or reduction in, pathogenic bacteria in both environmental contexts and human hosts [18,19]. Due to the emergence of antibiotic resistance, these viruses have gained renewed interest as potential alternatives to the treatment of multidrug-resistant (MDR) bacterial infections. In phage therapy, bacteriophages selectively target and destroy their host bacterium upon producing progeny phages, which offer advantages such as high specificity, no cytotoxic effects on eukaryotic cells, and minimal disruption to the microbiome [20]. However, it is constrained by a narrow host range, the development of phage resistance, and challenges in clinical formulation [21,22,23]. To overcome these challenges, several emerging approaches have been developed to enhance the therapeutic potential and clinical viability of phage-based treatment [24]. The narrow host range limitation of bacteriophages can be effectively addressed through several complementary strategies that enhance therapeutic efficacy and clinical applicability. Phage cocktails, consisting of multiple bacteriophage types, function analogously to combination drug therapies by targeting diverse bacterial strains present in clinical infections, while phage libraries consisting of comprehensive collections of characterized bacteriophages serve as expanded reserves, allowing precise matching against newly isolated target pathogens [25,26,27]. Additionally, extensive screening protocols utilizing broad host ranges can identify bacteriophages that exploit common surface receptors, enabling single phages to target multiple isolates across different pathogenic species, and genetic engineering approaches can further expand host range by modifying phage components responsible for host binding or incorporating additional host-binding proteins [28,29,30]. To address the emergence of bacteriophage-resistant bacterial strains, combination therapies utilizing both phages and conventional antibiotics have shown remarkable promise, as their distinct bactericidal and bacteriostatic mechanisms create synergistic effects that restore antibiotic sensitivity in resistant bacteria, reduce resistance development probability, and enhance efficacy against biofilm-associated infections where antibiotic tolerance is typically elevated [31,32,33]. Furthermore, endolysins, the peptidoglycan-degrading enzymes encoded by bacteriophages, offer distinct therapeutic advantages over whole-phage therapy by providing more predictable therapeutic outcomes and simplified quality assessment, with recent advances in endolysin engineering, including synthesis and transformation of lysin-coding genes into antimicrobial peptides, significantly enhancing antibacterial activity beyond that of native bacteriophage [34,35,36,37,38,39]. During the phage lytic cycle, endolysins facilitate the breakdown of peptidoglycan, enabling the release of progeny phages [40,41], as peptidoglycan is crucial for bacterial structural integrity and protection against osmotic stress; thus, its degradation results in bacterial lysis [42]. Moreover, endolysins are regarded as more advantageous antibacterial agents than entire phages, especially in clinical settings, owing to difficulties in phage quality control [43,44,45]. Endolysins often have a catalytic domain exhibiting enzymatic capabilities, including peptidase, amidase, or glycosidase activity, and may also include a substrate-binding domain, depending on their structural class and host origin [46]. Gram-positive bacteria are generally more vulnerable to endolysins because their peptidoglycan layer is directly exposed, whereas in Gram-negative bacteria, it is protected by an outer membrane [42]. 

The outer membrane of Gram-negative bacteria is a highly asymmetric bilayer, consisting of a phospholipid-rich inner leaflet and a lipopolysaccharide (LPS)-rich outer leaflet [47]. LPS is a structurally complex molecule comprising three distinct regions: lipid A, the core oligosaccharide, and the O-antigen. Lipid A, which anchors the LPS into the membrane, consists of a phosphorylated diglucoseamine backbone with four to seven acyl chains. It is covalently linked to the core oligosaccharide, a segment of 8–12 sugar residues typically categorized into inner and outer core regions. The core is highly phosphorylated and thus strongly anionic, whereas the O-antigen, composed of repeating sugar units, extends outward and forms a hydrophilic barrier at the bacterial surface [48,49,50,51]. This unique LPS architecture contributes significantly to the impermeability of the outer membrane. Hydrophilic macromolecules are hindered by the hydrophobic lipid bilayer, whereas hydrophobic substances encounter resistance due to the dense, charged sugar components of the LPS core and O-antigen [52]. Additionally, LPS molecules are stabilized by electrostatic interactions mediated by divalent cations, particularly magnesium (Mg^2+^) and calcium (Ca^2+^), which bridge negatively charged phosphate groups within the inner core. These cation-mediated interactions are crucial for maintaining membrane integrity and resistance to many antimicrobial agents [53]. Ethylenediaminetetraacetic acid (EDTA), a polyaminocarboxylic acid and potent chelator of divalent cations, disrupts these interactions by sequestering Mg^2+^ and Ca^2+^ ions from the LPS layer. This chelation weakens the outer-membrane structure, facilitates the release of LPS, and increases permeability, thereby enhancing bacterial susceptibility to various antimicrobials [54,55]. Beyond EDTA, other outer membrane permeabilizers (OMPs), including citric acid and triton X-100, have also been shown to disrupt the outer membrane and potentiate the activity of antimicrobial agents. Combining endolysins with such OMPs significantly improves their bactericidal efficacy against Gram-negative pathogens, overcoming the barrier function of the outer membrane and enabling enzymatic access to the peptidoglycan layer [56,57,58].

This study focused on the endolysin of *A. baumannii*-specific phage TAC1. Bacteriophage TAC1 [59], previously isolated from sewage water at the Department of Microbiology and Molecular Genetics, University of the Punjab, Pakistan, using an *A. baumannii* clinical isolate as the host bacterium, demonstrated a narrow host range, infecting 21 of 32 (66%) *A. baumannii* clinical isolates tested with marked lytic activity, while showing no activity against other bacterial species including *Klebsiella pneumonia*, *Pseudomonas aeruginosa*, *Staphylococcus aureus*, *Escherichia coli*, *Enterobacter cloacae*, *Burkholderia cepacia*, and *Serratia marcescens*. Genomic analysis revealed that phage TAC1 has a dsDNA genome size of 101.77 kb with 37.51% GC content and contains 161 predicted ORFs. Among these ORFs, TAC1 encodes an endolysin (ORF57) belonging to the glycoside hydrolase family, but, surprisingly, no conventional holin-encoding gene was identified in the TAC1 genome [60]. However, ORF55 was found to encode a hypothetical protein with an AMP-binding domain and three transmembrane domains, suggesting it may function as a novel, uncharacterized holin like those found in related *myoviridae* bacteriophages [61]. The endolysin ORF 57 sequence, designated as LysTAC1, was retrieved from NCBI and synthesized for this study. The cloning, expression, and activity analysis of LysTAC1 were performed under various conditions against a range of bacterial pathogens, including Gram-negative clinical isolates of *A. baumannii* and different strains of *E. coli*, as well as Gram-positive strains of *S. aureus* ATCC 29213 and *E. gallinarum* HCD 28-1.

## 2. Results

### 2.1. Molecular Identification and Antibiotic Susceptibility Pattern of A. baumannii Clinical Isolates

All clinical isolates used in this study were identified as *A. baumannii* through molecular characterization. PCR amplification revealed specific gene fragments for *rpoB* and *gluconolactonase* genes, with amplicon sizes of 1024 base pairs (bp) and 185 bp, respectively. These molecular markers conclusively confirmed the species identification of the *A. baumannii* clinical isolates (Supplementary Figure S1).

Antibiotic susceptibility testing by disk diffusion revealed an extensive multidrug resistance pattern among all eleven *A. baumannii* clinical isolates using standard clinical breakpoints (Supplementary Table S1). The isolates exhibited distinct resistance profiles across the ten antibiotics tested, with imipenem (carbapenem) demonstrating universal resistance (11/11, 100%), followed by high resistance rates for piperacillin (10/11, 90.9%) and ceftriaxone (8/11, 72.7%). Aminoglycoside resistance was prominent, with gentamicin and amikacin showing resistance in 5/11 (45.5%) and 7/11 (63.6%) isolates, respectively. Fluoroquinolone susceptibility varied considerably, with ciprofloxacin resistance observed in 5/11 isolates (45.5%) and levofloxacin resistance in 4/11 isolates (36.4%). Tetracycline class antibiotics demonstrated differential activities, with tetracycline and doxycycline showing resistance in 6/11 (54.5%) and 5/11 (45.5%) isolates, respectively, while minocycline resistance was detected in 7/11 isolates (63.6%). This comprehensive resistance profiling demonstrates the heterogeneous multi-drug resistance nature of these clinical *A. baumannii* isolates with notable carbapenem resistance prevalence, underscoring the critical need for alternative therapeutic interventions.

### 2.2. Bioinformatic Analysis of LysTAC1 

**Figure 1:**
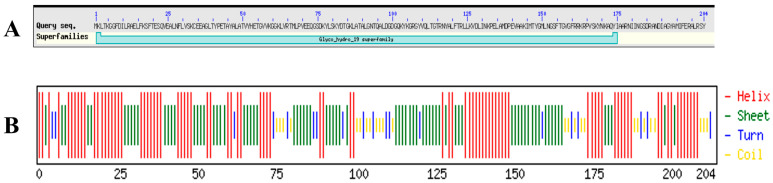
LysTAC1 properties. (**A**) BLASTp analysis of LysTAC1 amino-acid sequence showing glycoside hydrolase family 19 chitinase domain. (**B**) Predicted secondary structure of LysTAC1: H (helix) 126 residues (61.8%), E (beta sheet) 128 residues (62.7), and T (turn) 30 residues (14.7%).

**Figure 2:**
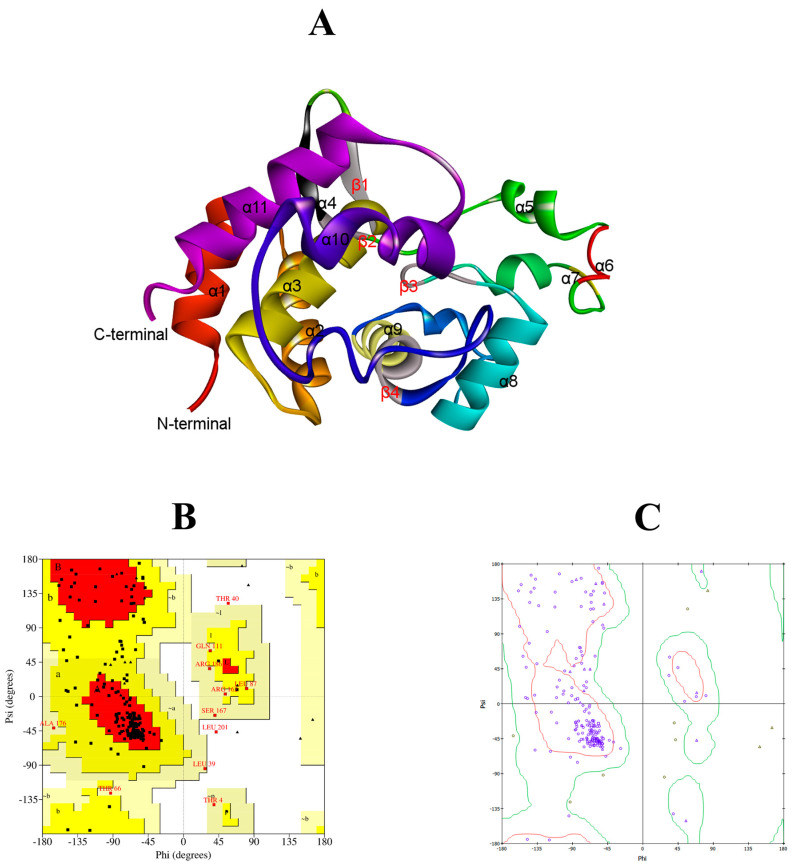
LysTAC1 tertiary structure analysis. (**A**) Predicted tertiary structure model of LysTAC1 showing α-helix and β-sheet regions. Ramachandran plots for the predicted tertiary structure of LysTAC1 generated by (**B**) PROCHECK and (**C**) Discovery studio. Plot statistics show residues in favored regions: 125 (70.6%), allowed regions: 50 (28.3%), and disallowed regions: 2 (1.1%).

**Figure 3:**
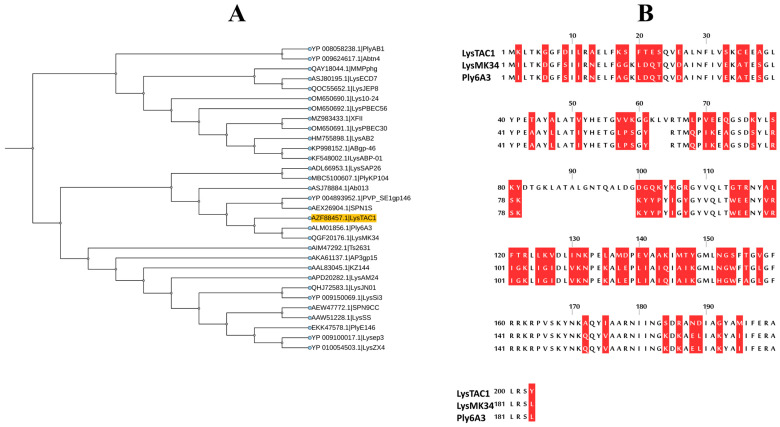
Phylogenetic analysis of LysTAC1 (**A**) Bootstrap consensus tree showing phylogenetic relationships of LysTAC1 and 30 previously reported Gram-negative endolysins. (**B**) Sequence Comparison of LysTAC1 (accession no. AZF88457.1), LysMK34 (accession no. QGF20176.1), and Ply6A3 (accession no. ALM01856.1) endolysins (red color indicates mutated residues).

The LysTAC1 protein consists of 204 amino acids, with an approximate molecular weight of 24 kDa. It features a glycoside hydrolase family 19 chitinase domain as predicted by the Conserved Domain Database (CDD) and Interpro scan (Figure 1A). Based on primary structural analysis, the protein has an isoelectric point (pI) of 9.42 and an instability index (II) of 29.09, indicating that LysTAC1 is a stable protein in bacterial systems. Additionally, no transmembrane domains or signal peptides were identified in the protein using the Deep TMHMM server. The secondary structure of LysTAC1 was predicted using the Chou and Fasman method (Figure 1B), and the protein is mainly made up of alpha-helix and beta-sheet regions, with turns and coils distributed throughout. A three-dimensional structural model of the LysTAC1 was generated in PDB format using the DI-TASSER server (Figure 2A), while its stability was analyzed using the Ramachandran plot (Figure 2B,C). Over 90% of the residues were found in the most favored and sterically allowed regions, indicating the high reliability and stability of the constructed model. Phylogenetic analysis with 30 previously reported Gram-negative endolysins revealed Ply6A3 and LysMK34 share a common ancestor with LysTAC1 (Figure 3A); however, further analysis via Clustal Omega v.1.2.4 showed a low level of similarity of LysTAC1 with Ply6A3 and LysMK34 (Figure 3B), suggesting the novelty of LysTAC1.

### 2.3. Molecular Docking of LysTAC1

**Figure 4:**
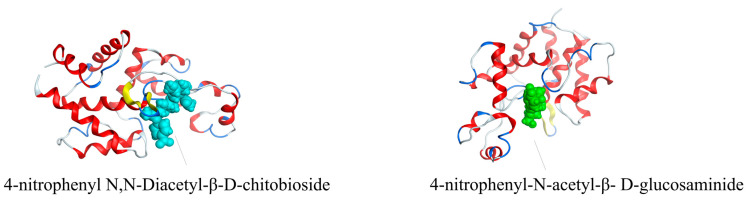
3D interaction of LysTAC1 with two chitinase substrates: 4-nitrophenyl N-acetyl-β-D-glucosaminide and 4-nitrophenyl N, N-Diacetyl-β-D-chitobioside.

The binding energy of 4-nitrophenyl N-acetyl-beta-D-glucosaminide and 4-nitrophenyl N, N-Diacetyl-beta-D-chitobioside to the LysTAC1 was estimated to be −5.82 kcal/mol and −6.85 kcal/mol, respectively (Figure 4). The observed negative binding energies substantiate the favorable molecular interaction and binding potential of the LysTAC1 with both ligands.

### 2.4. Over-Expression and Purification of LysTAC1

**Figure 5:**
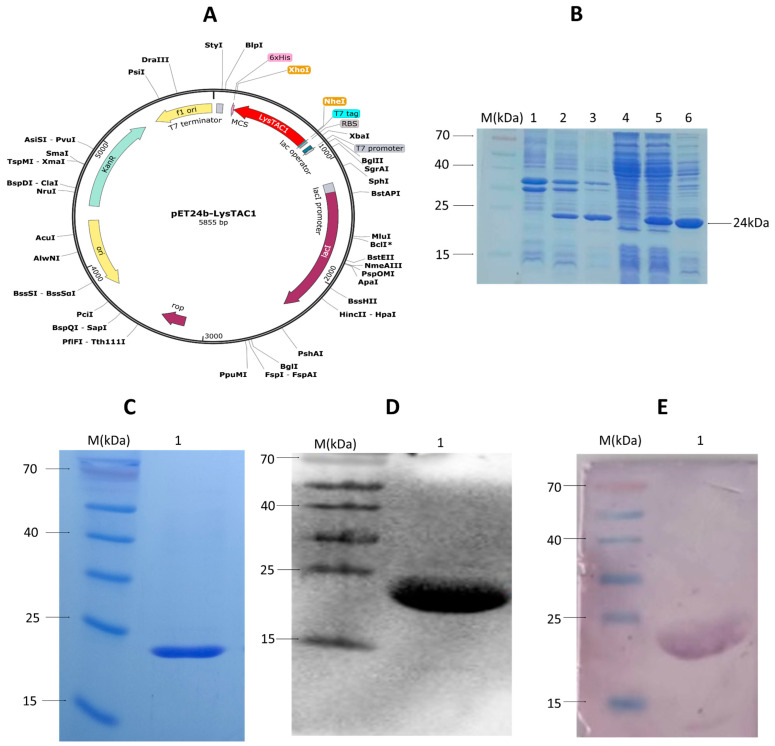
Recombinant plasmid pET-24b-LysTAC1 construction, expression optimization, and purification of LysTAC1. (**A**) Schematic diagram of recombinant plasmid construction. (**B**) SDS-PAGE analysis of LysTAC1 expression. Lane M: Page Ruler Prestained Protein Ladder; Lane 1: Pellet non-induced *E. coli* BL21 (Negative control); Lane 2–3: pellets induced with 0.5 mM and 1 mM IPTG; Lane 4: Supernatant of non-induced *E. coli* BL21 (Negative control); Lane 5–6: Supernatants induced with 0.5 mM and 1 mM IPTG. (**C**) Purified LysTAC1 on 15% SDS-PAGE staining with Coomassie blue staining. (**D**) Western blot using the ECL method; (**E**) Western blot using the colorimetric method. In panels (**C**–**E**): lane M: showing Page Ruler Prestained Protein Ladder; lane 1: purified 24 kDa LysTAC1.

The LysTAC1 gene was cloned into the pET-24b expression plasmid, and the recombinant protein (LysTAC1) with a C-terminal 6 × His-tag was expressed in *E. coli* BL21 (DE3). To determine the optimal induction condition for LysTAC1 expression, two IPTG concentrations (0.5 mM and 1 mM) were tested. No significant difference in the yield of soluble LysTAC1 protein was observed between the two conditions (Figure 5B). The purity and molecular weight of LysTAC1 (≈24 kDa) were confirmed by SDS-PAGE and Western blot analyses (Figure 5C–E).

### 2.5. Antibacterial Activity of LysTAC1

#### 2.5.1. Muralytic Activity

**Figure 6:**
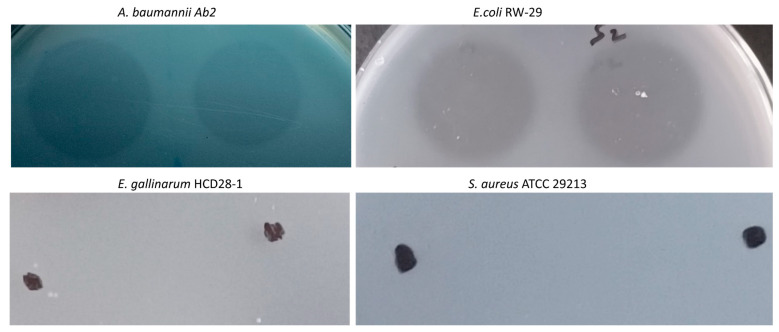
Muralytic activity of purified LysTAC1 against autoclaved *A. baumannii* Ab2 and *E. coli* RW-29, showing clear inhibition zones. No lysis was observed for *E. gallinarum* HCD 28-1 and *S. aureus* ATCC 29213; black dots indicate the spots where LysTAC1 was applied. 20 mMTris-HCl buffer (pH 7.0) was used as a negative control.

In the initial screening for muralytic activity, the purified LysTAC1 was tested using a plate-spot assay against *A. baumannii* Ab2, *E. coli* RW-29, *S. aureus* ATCC 29213, and *E. gallinarum* HCD 28-1 (Figure 6). The LysTAC1 exhibited strong cell-wall hydrolytic activity, as shown by the clear lysis zones on *A. baumannii* and *E. coli* lawns. These well-defined inhibition zones demonstrate the ability of LysTAC1 to efficiently degrade the cell walls of Gram-negative bacteria. In contrast, no zones of inhibition were observed on the lawns of *S. aureus* ATCC 29213 and *E. gallinarum* HCD 28-1, indicating that LysTAC1 does not exhibit muralytic activity against Gram-positive bacteria.

#### 2.5.2. LysTAC1 Antibacterial Activity with EDTA

**Figure 7:**
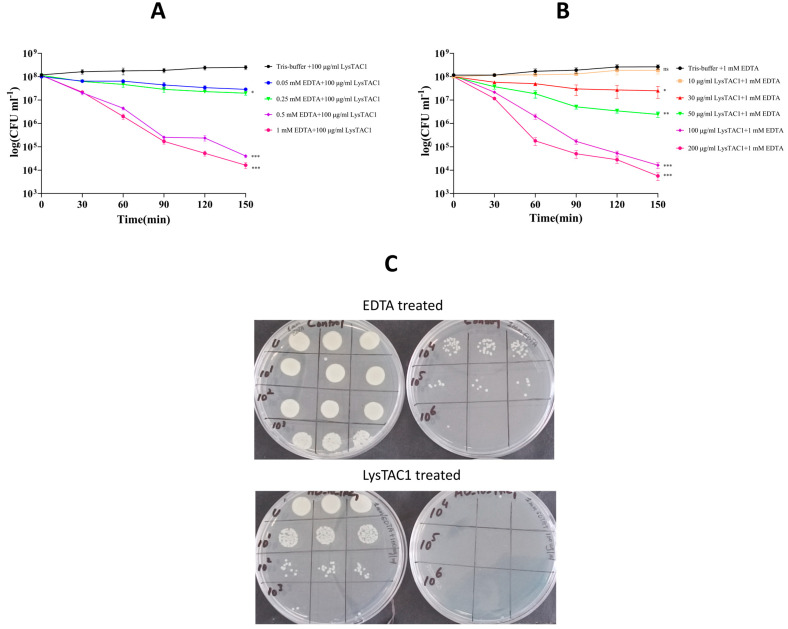
Concentration-dependent and EDTA-enhanced antibacterial activity of LysTAC1. (**A**) Effect of varying EDTA concentration with 100 μg/mL LysTAC1 against *A. baumannii* Ab2; LysTAC1 alone served control. (**B**) Effect of varying LysTAC1 concentrations with 1 mM EDTA against *A. baumannii* Ab2; 1 mM EDTA alone served as control; (**C**) CFU reduction in *A. baumannii* Ab2 following pretreatment with 1 mM EDTA and subsequent treatment with 100 μg/mL LysTAC1. Control plates (Top) show EDTA treatment alone; combined treatment (bottom) demonstrates significant CFU reduction. Data in panels A and B are expressed as mean ± standard deviation (SD) from three independent biological replicates (*n* = 3). Statistical significance was determined by one-way ANOVA. Significance levels: ns (non-significant, *p* > 0.05); * *p* ˂ 0.05; ** *p* < 0.01; *** *p* < 0.001.

LysTAC1 alone did not exhibit antibacterial activity against *A. baumannii* Ab2, but it showed effectiveness when used in combination with EDTA. After 2.5 h of treatment with 100 μg/mL LysTAC1 and 0.5 mM or 1 mM EDTA, the bacterial count decreased from 10^8^ to 10^4^ CFU/mL, indicating a 4 log reduction. This suggests that EDTA likely enhanced the permeability of the bacterial outer membrane, thereby facilitating the lytic action of LysTAC1 (Figure 7A).

#### 2.5.3. LysTAC1 Dose Response Activity

The antibacterial activity of LysTAC1 against *A. baumannii* Ab2 in combination with 1 mM EDTA displayed a concentration-dependent effect. No bacterial reduction was observed at a concentration of 10 μg/mL. However, at concentrations of 30, 50, 100, and 200 μg/mL, there was a 1, 2, 4, and 5 log reduction, respectively, after 2.5 h of incubation. Furthermore, 1 mM EDTA alone did not lead to a reduction in viable cell numbers (Figure 7B).

#### 2.5.4. LysTAC1 Efficacy Across Bacterial Growth Phases 

**Figure 8:**
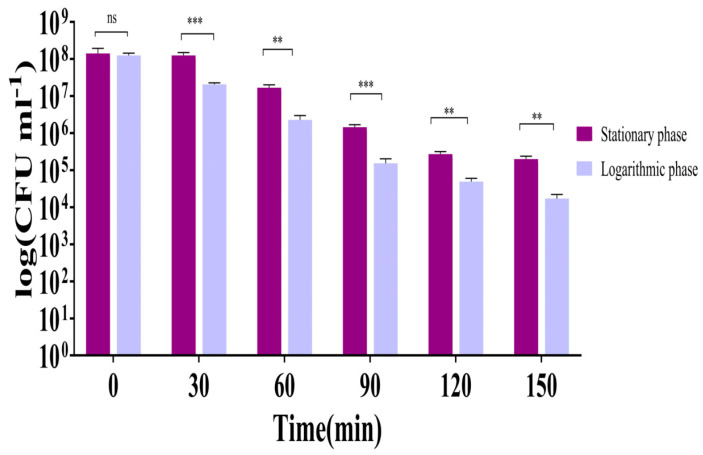
Growth phase-dependent antibacterial activity of LysTAC1. LysTAC1 demonstrated significant antibacterial activity against *A. baumannii* Ab2 in logarithmic phase with a gradual reduction in viable cell count over time, while showing minimal effect on stationary phase cells, indicating clear growth phase-dependent efficacy. Data are expressed as mean ± standard deviation (SD) from three independent biological replicates (*n* = 3). Statistical significance was determined by an unpaired *t*-test. Significance levels: ns (non-significant, *p* > 0.05); ** *p* < 0.01; *** *p* < 0.001.

The antibacterial effectiveness of LysTAC1 was assessed against *A. baumannii* Ab2 during both the stationary and logarithmic growth phases. The LysTAC1 showed much greater antibacterial activity during the logarithmic phase, indicating a stronger effect on actively dividing bacterial cells, while its activity was lower during the stationary phase (Figure 8).

#### 2.5.5. LysTAC1 Against Diverse Bacterial Strains

**Figure 9:**
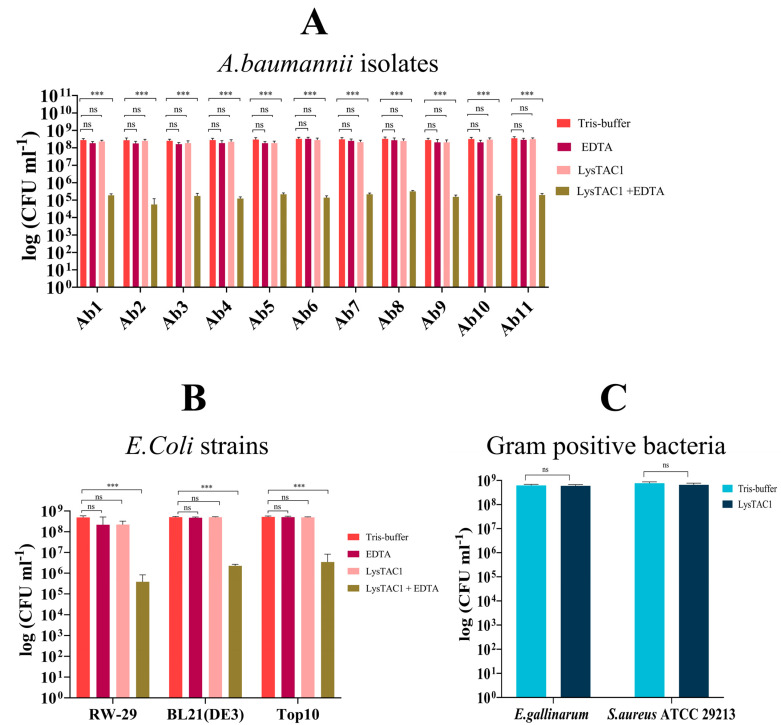
Antibacterial activity of LysTAC1 against diverse bacteria. (**A**) Activity against *A. baumannii* clinical isolates (Ab1-Ab11) showing bacterial counts (CFU/mL) after 2.5 h of treatment with Tris-HCl buffer (Control), EDTA, LysTAC1 alone, and LysTAC1 + EDTA combination. (**B**) Activity against *E. coli* strains under identical treatment conditions. (**C**) Activity against Gram-positive bacteria after 2.5 h of treatment with Tris-HCl buffer (control) and LysTAC1. Data are expressed as mean ± standard deviation (SD) from three independent biological replicates (*n* = 3). Statistical significance was determined by one-way ANOVA (in panels A and B) and unpaired *t*-test (in panel C). Significance levels: ns (non-significant, *p* > 0.05); *** *p* < 0.001.

LysTAC1 exhibited distinct antimicrobial activity profiles against diverse bacterial strains, revealing critical insights into its spectrum of action and membrane-dependent efficacy mechanisms. Against eleven clinical isolates of carbapenem-resistant *A. baumannii* (Ab1–Ab11), LysTAC1 monotherapy demonstrated no significant bactericidal activity, with bacterial viability remaining 10^8^ CFU/mL comparable to 20 mM Tris-HCl buffer (pH 7.0) controls. However, outer membrane permeabilization with 1 mM EDTA dramatically enhanced LysTAC1 efficacy, achieving substantial bacterial reduction to approximately 10^4^–10^5^ CFU/mL, representing 3–4 log reduction in viable counts (Figure 9A). This membrane-dependent activity pattern was consistently observed across *E. coli* strains, where LysTAC1 alone failed to reduce bacterial loads, but EDTA pretreatment facilitated significant LysTAC1-mediated killing (Figure 9B). In contrast, Gram-positive bacteria *S. aureus* ATCC 29213 and *E. gallinarum* HCD 28-1, despite the absence of outer-membrane barriers, exhibited complete resistance to LysTAC1 treatment (Figure 9C).

#### 2.5.6. TEM of LysTAC1-Treated *A. baumannii*

**Figure 10:**
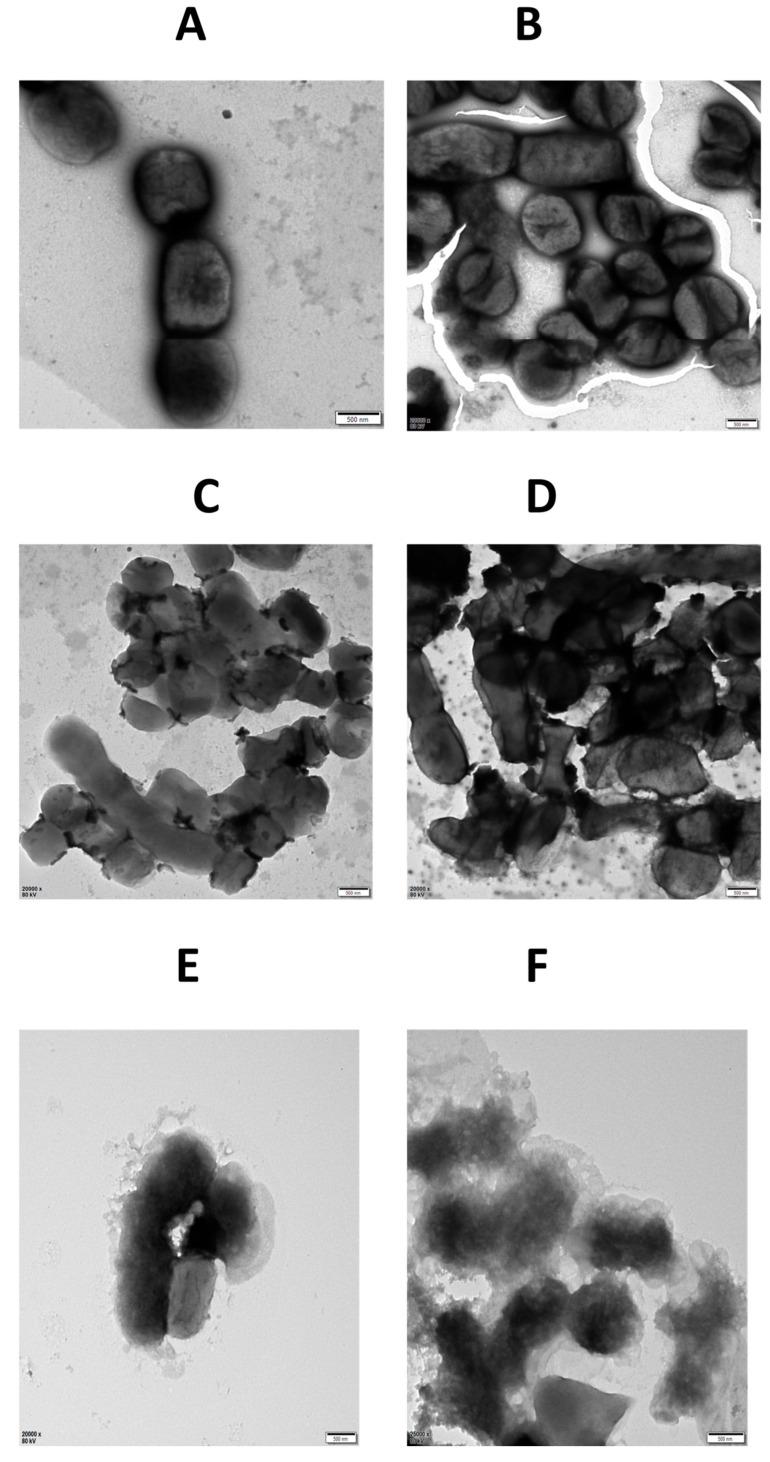
Transmission electron micrographs of *A. baumannii* Ab2. (**A**,**B**) Untreated log phase cells; (**C**,**D**) 1 mM EDTA treatment; (**E**,**F**) 100 μg/mL LysTAC1 treatment following EDTA exposure. Scale bar = 500 nm.

Transmission electron microscope (TEM) was used to visualize the structural effects of LysTAC1 on *A. baumannii* Ab2 cells. Untreated control cells appeared intact with smooth outer membranes, indicating preserved cellular integrity (Figure 10A,B). Treatment with 1 mM EDTA caused partial outer-membrane disruption, characterized by irregular membrane contours and slight loosening consistent with lipopolysaccharide (LPS) removal, while overall cellular structure remained largely intact (Figure 10C,D). Conversely, cells exposed to both 1 mM EDTA and 100 μg/mL LysTAC1 exhibited extensive morphological damage, including complete cell envelope disintegration and cytoplasmic leakage (Figure 10E,F).

### 2.6. Stability of LysTAC1

**Figure 11:**
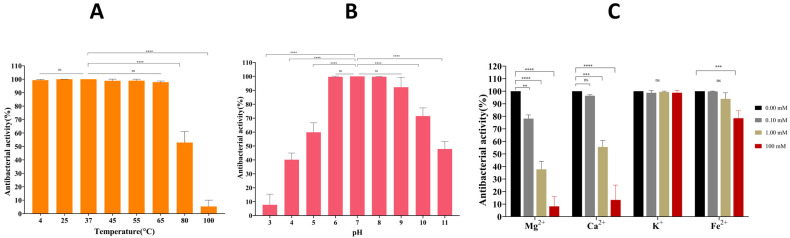
Effect of environmental factors on LysTAC1 lytic activity against *A. baumannii* after 2.5 h of treatment. (**A**) Temperature stability after 1 h pre-incubation; (**B**) pH stability after 1 h pre-incubation; (**C**) metal ion concentration. Data are expressed as mean ± standard deviation (SD) from three independent biological replicates (*n* = 3). Statistical significance was determined by one-way ANOVA. Significance levels: ns (non-significant, *p* > 0.05 ); ** *p* < 0.01; *** *p* < 0.001; **** *p* < 0.0001.

LysTAC1 activity remained stable after 1-h treatment at 4, 25, 37, 45, 55, and 65 °C. However, activity decreased to approximately 50% at 80 °C and to less than 10% at 100 °C (Figure 11A). This thermal stability indicates LysTAC1’s potential suitability for temperature-variable applications. LysTAC1 activity remained unaffected at pH 6.0–8.0. At pH 3.0, 4.0, 5.0, 9.0, 10, and 11, activity was reduced to approximately 7%, 40%, 60%, 92%, 71%, and 47%, respectively (Figure 11B). LysTAC1 stability varied depending on metal ion type and concentrations (Figure 11C). Mg^2+^ demonstrated the strongest inhibitory effect, reducing activity to 78%, 38%, and 8% at 0.1, 1.0, and 100 mM, respectively. Ca^2+^ showed similar dose-dependent inhibition, maintaining 97% activity at 0.1 mM but decreasing to 58% and 14% at 1.0 and 100 mM, respectively. K^+^ ions had no significant effect across all concentrations (99–100% activity retained), while Fe^2+^ remained non-inhibitory at 0.1 and 1.0 mM (100% and 95% activity) but reduced activity to 79% at 100 mM.

## 3. Discussion

Multidrug-resistant (MDR) Gram-negative infections, especially those caused by carbapenem-resistant *A. baumannii*, pose a substantial global healthcare burden, particularly in hospitalized patients [62]. In this study, antibiotic susceptibility testing demonstrated an extensive multidrug resistance pattern in all eleven *A. baumannii* clinical isolates, with universal imipenem resistance (100%) and a high resistance rate to β-lactams (72.7–90.9%). The variable susceptibility to aminoglycosides, fluoroquinolones, and tetracyclines underscores the heterogenous nature of resistance mechanisms in these clinical strains. Our findings of extensive multidrug resistance align with previous large-scale studies from China. A comprehensive study of 27,754 *A. baumannii* strains reported a high resistance rate across multiple antibiotic classes, with notable variation based on patient demographics and infection sites, particularly demonstrating high carbapenem resistance rates among male patients in the 15–50-year age group [63]. Similarly, another study from China reported that 50 multidrug-resistant *A. baumannii* isolates showed 92% carbapenem resistance along with high resistance rates to multiple antibiotic classes [64]. The Study for Monitoring Antimicrobial Resistance Trends (SMART) ongoing surveillance initiative (2011–2014) across six global regions reported high antimicrobial resistance, with no single drug inhibiting >70% of *A. baumannii* isolates globally, and imipenem susceptibility ranging from 64% in North America to ≤11% in Europe and the Middle East [65]. Additionally, a Lebanese tertiary hospital reported 81% prevalence of carbapenem-resistant *A. baumannii* in 2018 [66]. These consistent findings across different geographical regions and study populations confirm the global nature of multidrug resistance in *A. baumannii*, emphasizing the urgent need for alternative therapeutic strategies beyond conventional antibiotics. 

Bacteriophage-derived endolysin has emerged as a promising alternative to conventional antibiotics for addressing multidrug-resistant infections [67,68]. In this study, we successfully synthesized, cloned, overexpressed, and characterized a novel endolysin, LysTAC1, derived from an *A. baumannii*-specific lytic phage, TAC1 [59]. Conserved domain analysis indicated that LysTAC1 has a glycoside hydrolase family 19 chitinase domain, homologous to the previously characterized endolysins Abtn4 and SPN1S [69,70].

Phylogenetic analysis revealed that LysTAC1 shares a common evolutionary lineage with the *A. baumannii*-specific phage-derived endolysins Ply6A3 and LysMK34 [23,71]. Nonetheless, nucleotide sequence alignment demonstrated minimal resemblance, affirming the distinctiveness of LysTAC1 (Figure 3). Given the presence of a chitinase domain in LysTAC1, its interaction with chitinase substrates was assessed via molecular docking using MOE (Molecular Operating Environment). Docking simulations with 4-nitrophenyl N-acetyl-β-D-glucosaminide and 4-nitrophenyl N, N-Diacetyl-β-D-chitobioside yielded binding energies of −5.82 kcal/mol and −6.85 kcal/mol, respectively (Figure 4). To our knowledge, no prior studies have reported molecular docking of a chitinase domain-containing endolysin with chitinase substrates. However, a previous study employed molecular docking and molecular dynamics simulations to evaluate the antifungal activity of an amidase_2 domain-containing bacteriophage endolysin, reporting binding free energies of −5.6 kcal/mol for the amidase_2 domain-chitin interaction [72]. Muralytic activity assays performed on autoclaved *A. baumannii* and *E. coli* cultures demonstrated that LysTAC1 formed clear zones of clearance (Figure 6), confirming its ability to degrade bacterial peptidoglycan. Similar methodologies have been employed in previous studies to assess muralytic activity in endolysins such as Abp013, LysVTh2, and ElyA1 [73,74,75]. The antibacterial efficacy of LysTAC1 was further evaluated against 11 clinical isolates of *A. baumannii* and pathogenic (RW-29) and genetic (BL21, Top10) strains of *E. coli* (Figure 9A,B). When applied alone, LysTAC1 did not exhibit an antibacterial effect, presumably due to the inability to penetrate the outer membrane of Gram-negative bacteria [76]. To address this constraint, bacterial cells were pretreated with different concentrations of EDTA (0.05 mM, 0.25 mM, 0.5 mM, and 1 mM) to rupture the outer membrane. A significant reduction (≈3–4 log) in bacterial counts was observed when cells were treated with 0.5 mM or 1 mM EDTA followed by exposure to 100 µg/mL of LysTAC1, whereas EDTA alone had no bactericidal effect. Additionally, LysTAC1 exhibited concentration-dependent antibacterial activity, with bacterial reductions of 1 log, 2 log, and 5 log at concentrations of 30 µg/mL, 50 µg/mL, and 200 µg/mL, respectively (Figure 7). These findings align with prior studies that reported Gram-negative endolysins, such as PlyAB1, Lys68, LysZX4, and XFII, exhibiting antibacterial efficacy in the presence of outer membrane permeabilizer [77,78,79,80]. In addition, we evaluated the activity of LysTAC1 against Gram-positive bacteria such as *S. aureus* ATCC 29213 and *E. gallinarium* HCD 28-1, but no antibacterial effect was observed (Figure 9C). This is consistent with previous endolysins, such as LysKP213 and SPN9CC, from Gram-negative backgrounds, which show specificity to Gram-negative bacteria only, likely due to differences in cell-wall structure and accessibility [81,82]. Our findings underscore the use of membrane-disrupting agents in augmenting the therapeutic efficacy of endolysins against Gram-negative bacteria. It is well established that EDTA functions by chelating divalent cations that stabilize the bacterial outer membrane, thus enabling endolysin penetration to the peptidoglycan layer. Nonetheless, the clinical utilization of EDTA is limited by its coagulation characteristics [83]. Citric acid and malic acid have been suggested as more appropriate alternatives for in vivo endolysin-based antibacterial therapy [78]. In addition to chemical permeabilizers, peptide modifications, such as membrane-penetrating peptides, membrane-translocating domains, and polycationic nonpeptides, have demonstrated the ability to augment endolysin effectiveness against Gram-negative bacteria [56,83,84]. Notably, Art-TOP3, an endolysin fused to cathelicidin antimicrobial peptide (CAMP) for outer-membrane destabilization, demonstrated potent in vitro efficacy against carbapenem-resistant *A. baumannii* clinical isolates and ATCC 19606 strain, reducing bacterial counts by ≥5 log within one hour. Time kill analysis using *A. baumannii* ATCC 19606 revealed rapid, dose-dependent killing with ≥6 log reduction within 1–10 min at 10–100 μg/mL. In vivo efficacy was further confirmed in *Galleria mellonella* larvae infected with MDR *A. baumannii*, where treatment with 250–500 μg/mL Art-Top3 achieved 87–92% larval survival at 24 h compared to 23% in untreated controls [38]. Similarly, N-terminal fusion of cecropin A to endolysin LysMK34, creating the engineered variant eLysMK34, substantially enhanced antimicrobial efficacy, reducing bacterial counts by >4–5 logs within 30 min for both colistin-sensitive and resistant *A. baumannii* strains, compared to the unmodified LysMK34, which required 120 min to achieve 3–4 log reduction [39]. Additionally, fusion of the membrane-permeabilizing peptide CeA to endolysin LysAB2 resulted in up to a 100,000-fold increased activity against multidrug-resistant *A. baumannii* and enhanced capability to disrupt biofilm formation [85]. Furthermore, fusion of the membrane-penetrating peptide DS4.3 to endolysin PA90 significantly enhanced its bactericidal activity against multidrug-resistant *A. baumannii* at low concentration [86]. 

The antibacterial efficacy of LysTAC1 was also affected by bacterial growth phase. Enhanced efficacy was shown against *A. baumannii* during the logarithmic growth compared to the stationary phase (Figure 8). This finding is consistent with previously reported endolysins PlyPa01, Plypa96, and PlyF307, indicating that structural and compositional alterations in the outer membrane and peptidoglycan throughout different growth phases influence susceptibility differences [87,88]. Thermal stability analysis revealed that LysTAC1 remained functional at temperatures up to 65 °C, retained around 50% activity following exposure to 80 °C, and exhibited less than 10% residual activity at 100 °C (Figure 11A). Most reported endolysins exhibit optimal temperature stability within the range of 30–40 °C, with some exhibiting stability at elevated temperatures of 65 to 80 °C [89,90,91,92], and, in exceptional cases, up to 90 °C for brief durations [93]. Notably, LysBT1 demonstrates exceptional thermostability, retaining more than 60% activity after 1 h incubation at 95 °C, which represents remarkably high thermal resistance for an endolysin [94]. pH stability assays demonstrated that LysTAC1 retained significant activity within a pH range of 6.0–9.0, with reduced activity observed at extreme pH values. Specifically, activity was diminished to 7%, 40%, 60%, 71%, and 47% at pH 3.0, 4.0, 5.0, 10, and 11, respectively (Figure 11B). While most endolysins have been reported to function optimally within pH 5–10 [57,77,89], a few have been documented to remain active up to pH 11–12 [81,94,95]. The stability and antibacterial activity of LysTAC1 were also influenced by the presence and concentration of metal ions. Mg^2+^ at 0.1 mM, 1 mM, and 100 mM significantly reduced enzymatic activity, as did Ca^2+^ at 1 mM and 100 mM. In contrast, Fe^3+^ at 0.1 mM and 1 mM had no effect, but a marked reduction in activity was observed at 100 mM. Interestingly, K^+^ had no impact on enzymatic activity across all tested concentrations (0.1 mM, 1 mM, and 10 mM), suggesting tolerance to this ion (Figure 11C). The stability profile of LysTAC1 in the presence of metal ions closely resembled that of the previously characterized endolysin LysZX4-NCA [77]. There is considerable variability in the stability of different endolysins in response to metal ions, as reported in prior studies [96,97,98]. The influence of metal ions on endolysin activity is highly dependent on factors such as ion type, concentration, and enzyme structural properties. These findings highlight the need to consider ionic conditions when optimizing endolysins for therapeutic applications. This study has several limitations. First, LysTAC1 efficacy was evaluated only in vitro; in vivo validation in infection models is essential to assess therapeutic potential, pharmacokinetics, and toxicity. Second, while EDTA enhanced activity, its cytotoxic and anticoagulant properties limit its clinical utility. Future studies should explore safer permeabilizers or engineered peptide fusions for enhanced intracellular delivery. Finally, although docking results suggest substrate binding, experimental confirmation of chitinase activity through biochemical assays would strengthen mechanistic claims.

## 4. Materials and Methods

### 4.1. Strains and Culture Conditions

This study utilized 11 clinical isolates of *A. baumannii* (Ab1–Ab11) collected from the Microbiology Laboratory of Central Hospital of Dalian University of Technology, China, in January 2023, with all isolates originating from sputum samples of different patients. Additionally, pathogenic strains of *E. coli* RW-29, *S. aureus* ATCC 29213, and *E. gallinarum* HCD 28-1 were from the laboratory repository of the Department of Biochemistry and Molecular Biology, Dalian Medical University. For Cloning, the pET-24b vector, *E. coli* strains Top10, and BL21 (DE3) were employed (Table 1). *A. baumannii* isolates, *E. coli* strains (both genetic and pathogenic), and *E. gallinarum* HCD 28-1 were cultured in Luria–Bertani (LB) broth at 37 °C with shaking at 180 rpm. For solid media, LB agar (1% agar) was used, while transformation assays were performed using kanamycin-supplemented LB medium (50 μg/mL). *S. aureus* ATCC 29213 was cultured in Gifu anaerobic medium (GAM) liquid medium (peptone (10 g/L), soybean peptone (3 g/L), yeast extract powder (5 g/L), beef extract powder (2.2 g/L), digested serum powder (13.5 g/L), beef liver extract powder (1.2 g/L), glucose (3 g/L), potassium dihydrogen phosphate (2.5 g/L), NaCl(3 g/L), soluble starch (5 g/L), L-cysteine(0.3 g/L), and sodium thioglycolate (0.3 g/L) under identical conditions, with nutrient agar (tryptone (10 g/L), beef extract (4 g/L), NaCl (5 g/L), and agar (15 g/L) for solid media cultivation.

### 4.2. Molecular Identification and Antibiotic Susceptibility of A. baumannii Clinical Isolates

Molecular identification of *A. baumannii* clinical isolates was achieved by amplifying of *rpoB* and *gluconolactonase* genes [100], using primers listed in (Table 1). Genomic DNA was extracted using the boiling method [101], which entails suspending a full loop of bacterial isolates in 300 µL of distilled water and subsequently boiling for 10 min. After centrifugation at 6000 rpm for 5 min, the supernatant containing the DNA template was employed in the polymerase chain reaction (PCR) assay. The sequence-specific PCR assay for *rpoB* amplification included an initial denaturation at 97 °C for 15 min, followed by 30 cycles at 95 °C for 1 min, annealing at 57 °C for 45 s, and extension at 72 °C for 1 min, with a final extension at 72 °C for 10 min. However, the PCR protocol for the *gluconolactonase* gene comprised an initial denaturation at 95 °C for 5 min, followed by 30 cycles of denaturation at 95 °C for 1 min, annealing at 56.5 °C for 45 s, and extension at 72 °C for 1 min, with a final extension at 72 °C for 10 min. The PCR products were electrophoresed using 2% agarose gel.

Antimicrobial susceptibility profiles of *A. baumannii* clinical isolates were determined using the standardized Kirby–Bauer disk diffusion method. Bacterial suspensions adjusted to 0.5 McFarland turbidity standard were uniformly spread on Mueller–Hinton agar plates [102]. The antimicrobials tested included the following: piperacillin (100 µg), ceftriaxone (30 µg), imipenem (10 µg), amikacin (30 µg), gentamicin (10 µg), tetracycline (30 µg), doxycycline (30 µg), minocycline (30 µg), ciprofloxacin (5 µg), and levofloxacin (5 µg) (BKMAM, Changde, China). Antibiotic disks were placed and incubated at 37 °C for 24 h. Inhibition zones were measured and interpreted according to CLSI guidelines [103]. The interpretive criteria usedmin for *A. baumannii* were as follows: piperacillin ≥ 21 mm susceptible (S), 18–20 mm intermediate (I), ≤17 mm resistant (R); ceftriaxone ≥ 21 mm (S), 14–20 mm (I), ≤13 mm (R); imipenem ≥ 22 mm (S), 19–21 mm (I), ≤18 mm (R); amikacin ≥ 17 (S), 15–16 mm (I), ≤14 mm (R); gentamicin ≥ 15 mm (S), 13–14 mm (I), ≤12 mm (R); tetracycline ≥ 15 mm (S), 12–14 mm (I), ≤11 mm (R); doxycycline ≥ 13 mm (S), 10–12 mm (I), ≤9 mm (R); minocycline ≥ 16 mm (S), 13–15 mm (I), ≤12 mm (R); and ciprofloxacin ≥ 12 mm (S), 16–20 mm (I), ≤15 mm (R), and levofloxacin ≥ 17 mm (S), 14–16 mm (I), ≤13 mm (R).

### 4.3. Identification and Sequence Analysis of LysTAC1

The LysTAC1 endolysin gene from the *A. baumannii*-specific phage TAC1 (Accession number AZF88457.1) was retrieved from the National Centre for Biotechnology Information (NCBI) (https://www.ncbi.nlm.nih.gov/, accessed on 25 December 2022). The amino acid sequence of LysTAC1 was examined using the NCBI Basic Local Alignment Search Tool (BLASTp, https://blast.ncbi.nlm.nih.gov/Blast.cgi, accessed on 25 December 2022), whereas its functional domain was assessed through the NCBI Conserved Domain Database (CDD) https://www.ncbi.nlm.nih.gov/Structure/cdd/wrpsb.cgi, accessed on 26 December 2022) and Interpro scan (https://www.ebi.ac.uk/interpro/, accessed on 26 December 2022). Multiple sequence alignment and phylogenetic analysis were carried out with Clustal Omega (https://www.ebi.ac.uk/jdispatcher/msa/clustalo, accessed on 10 February 2023) and IQ-TREE v.2.3.6. The primary structural characteristics of LysTAC1 were analyzed using Protparam (https://web.expasy.org/protparam/, accessed on 10 February 2023), and transmembrane domain prediction was conducted with Deep TMHMM (https://dtu.biolib.com/DeepTMHMM/, accessed on 10 February 2023). Secondary structure was predicted via the Chou and Fasman approach (https://www.biogem.org/tool/chou-fasman/index.php, accessed on 2 March 2023). The tertiary structure was constructed in PDB format using the D-I-TASSER server (https://www.aideepmed.com/D-I-TASSER/help.html, accessed on 5 March 2023). The developed structural model was examined with Discovery Studio Visualizer v.21.1.0.20298. For the assessment of model stability, the 3D PDB model was analyzed using a Ramachandran plot via PROCHECK (https://saves.mbi.ucla.edu/, accessed on 25 March 2023) and Discovery Studio visualizer. 

### 4.4. Molecular Docking of LysTAC1

The endolysin LysTAC1, identified as a chitinase, was examined for its enzymatic interactions with two chitinase substrates: 4-nitrophenyl N, N-Diacetyl-beta-D-chitobioside (PubChem CID 10907699) and 4-nitrophenyl N-acetyl-beta-D-glucosaminide (PubChem CID 102416). Molecular docking was performed using MOE (Molecular Operating Environment) software v.2022.02 to predict the interaction.

### 4.5. Synthesis, Molecular Cloning, and Over-Expression of LysTAC1 

The endolysin LysTAC1 gene (615 bp) was synthesized without a stop codon and with NheI and XhoI restriction sites (total length 624 bp). The gene was integrated into the pET-24b plasmid between NheI and XhoI sites with a C-terminal 6 × His-tag by (Novagen, Madison, WI, USA). Restriction digestion and sequence analysis confirmed correct cloning. The resulting recombinant plasmid pET-24b-LysTAC1 was transformed into *E. coli* BL21 (DE3).

LysTAC1 was overexpressed in *E. coli* BL21 (DE3) carrying pET-24b-LysTAC1. Log phase cultures (OD_600_ ≈ 0.5) were induced with 1 mM isopropyl β-D-thiogalactopyranoside (IPTG) and grown to OD_600_ ≈ 1.0. Cells were harvested (5000× *g*, 10 min, 4 °C) and resuspended in lysis buffer (50 mM Tris-HCl, pH 8.0, 100 mM NaCl, 5% glycerol, 0.1 mM EDTA, 100 mM PMSF). The suspension was sonicated (pulse 40%, time 15 min, cycle 30 s) using a Vibra-Cell Sonicator (SONICS & MATERIAL Inc., Newtown, CT, USA) on ice until a clear lysate was obtained. Subsequently, the lysate was centrifuged at 4000× *g* at 4 °C for 10 min to remove un-lysed bacterial cells, followed by centrifugation at 17,300× *g* at 4 °C for 10 min. Supernatants and pellets were obtained and analyzed using 15% SDS-PAGE followed by staining with Coomassie Brilliant Blue R-250.

### 4.6. Western Blot Analysis 

Western blot analysis was carried out as previously reported [104,105], with some modifications. Two independent Western blot assays were conducted utilizing different detection methods. Cell lysates expressing LysTAC1 were subjected to 15% SDS-PAGE and subsequently transferred to nitrocellulose membranes. Following a 2-h incubation with 5% skimmed milk at room temperature, the membranes were rinsed with TBST buffer (NaCl, 20 mM Tris-HCl, pH 8.0, Tween 20). Then, membranes were incubated with α(anti)-polyhistidine clone HIS-1 (1:3000 dilution) (Sigma, Burlington, MA, USA) for 12 h at 4 °C. After three washes with TBST, one membrane was incubated for 2 h at room temperature with horseradish peroxidase (HRP)-conjugated goat anti-mouse antibodies (1:3000 dilution) (Proteintech Group, Inc., Rosemont, IL, USA) for enhanced chemiluminescence (ECL) detection. Reagents from the Western blotting detection kit (Advansta Inc., Sane Jose, CA, USA) were applied to a nitrocellulose membrane, and the LysTAC1-specific band was detected using an Image Lab equipment (BioRad, Hercules, CA, USA). The second membrane was incubated at room temperature for 2 h with alkaline phosphatase (AP)-conjugated goat anti-mouse antibodies (1:3000 dilution) (Proteintech, Group, Inc., Rosemont, IL, USA) for colorimetric visualization. The 5-Bromo-4-chloro-3-indolyl phosphate (BCIP), Nitro blue tetrazolium (NBT), and Western blot development buffer (100 mM Tris-HCl, pH 9.5, 100 mM NaCl, 5 mM MgCl_2_) were applied to the membrane, and the LysTAC1 band was analyzed. 

### 4.7. LysTAC1 Purification

LysTAC1 was purified using Nickel-nitrilotriacetic acid (Ni-NTA) affinity chromatography (QIAGEN, Hilden, Germany) following the manufacturer’s instructions. For further purification, LysTAC1 was processed through a Microsep advanced centrifugal device with 10 kDa MWCO (PALL Corporation, Port Washington, NY, USA). Protein purity was assessed by 15% SDS-PAGE followed by Coomassie Brilliant Blue R-250 staining. LysTAC1 concentration was determined using the bicinchoninic acid (BCA) assay kit (TIANGEN Biotechnology, Beijing, China).

### 4.8. Antibacterial Characterization of LysTAC1

#### 4.8.1. Muralytic Activity

Muralytic activity of LysTAC1 was examined using the plate lysis assay [73,75]. Clinical isolate *A. baumannii* Ab2, *E. coli* RW-29, *S. aureus* ATCC 29213, and *E. gallinarum* HCD 28-1 were cultivated to mid-logarithmic phase in LB or GAM media, pelleted, and resuspended in 20 mM Tris-HCl buffer (pH 7.0). Agar (0.8% *w/v*) was added to the bacterial suspension, autoclaved for 15 min at 120 °C, and then solidified in Petri plates. LysTAC1 (100 μg/mL) or 20 mM Tris-HCl buffer (as a negative control) was applied to the surface and incubated at room temperature for lysis zone observation. 

#### 4.8.2. LysTAC1 Antibacterial Activity with EDTA

The effect of the outer membrane permeabilizer (EDTA) on LysTAC1 antibacterial efficacy was assessed as described [77], with modifications. Logarithmic phase *A. baumannii* Ab2 was centrifuged and resuspended in 20 mM Tris-HCl buffer (pH 7.0) to 1 × 10^8^ CFU/mL. Bacterial cells were treated with EDTA (0, 0.05, 0.25, 0.5, and 1 mM) for 30 min, then centrifuged and washed twice to remove EDTA. Pre-treated bacteria were combined with LysTAC1 (final concentration 100 μg/mL) in 96-well plates (total volume 200 μL per well), with untreated bacteria as a control. Each condition was tested in triplicate wells and incubated at 37 °C for 2.5 h. Bacterial counts were assessed every 30 min by the plating method. Three independent biological replicates were performed for the entire experiment. 

#### 4.8.3. LysTAC1 Dose Response Activity

The concentration-dependent effect of LysTAC1 was investigated according to the protocol described by [69,77], with modifications. *A. baumannii* Ab2 (1 × 10^8^ CFU/mL) was pretreated with 1 mM EDTA and resuspended in 20 mM Tris-HCl buffer (pH 7.0). Various LysTAC1 final concentrations (10, 30, 50, 100, and 200 μg/mL) were mixed with pretreated bacterial suspension in 96-well plates (total volume 200 μL per well), with pretreated *A. baumannii* Ab2 without LysTAC1 serving as a control. Each concentration was tested in triplicate wells and incubated at 37 °C for 2.5 h. Bacterial counts were determined by standard plating at 30-min intervals. Three independent biological replicates were performed for the entire experiment. 

#### 4.8.4. LysTAC1 Efficacy Across Bacterial Growth Phases

The antibacterial activity of LysTAC1 was tested against *A. baumannii* Ab2 during both logarithmic phase (OD_600_ ≈ 0.6) and stationary phase (OD_600_ ≈ 1.6) by following the method reported by [106], with modifications. Briefly, in 96-well plates, bacterial culture (1 × 10^8^ CFU/mL) pre-treated with 1 mM EDTA and LysTAC1 at a final concentration of 100 μg/mL were mixed, maintaining a total volume of 200 μL per well. Followed by incubation at 37 °C for 2.5 h, bacterial populations were quantified at 30-min intervals. The entire experiments were repeated three times as independent biological replicates; each was conducted in triplicate wells.

#### 4.8.5. LysTAC1 Against Diverse Bacterial Strains

The antibacterial efficacy of LysTAC1 was determined against a diverse panel of bacterial strains, including carbapenem-resistant *A. baumannii* isolates, pathogenic (RW-29) and genetic (BL21 and Top10) strains of *E. coli*, *S. aureus* ATCC 29213, and *E. gallinarum* HCD 28-1. Logarithmic-phase bacterial cultures were prepared at a final concentration of 1 × 10^8^ CFU/mL. For Gram-negative strains, assays were conducted both with and without 1 mM EDTA pretreatment to enhance outer-membrane permeability. In contrast, Gram-positive strains were tested without EDTA treatment. LysTAC1 was introduced at a final concentration of 100 μg/mL in 96-well plate assays, maintaining a total volume of 200 μL per well. Following incubation at 37 °C for 2.5 h, viable bacterial counts were determined using standard plating methods to assess bacteriolytic activity. All experiments were performed in three independent biological replicates; each was conducted in triplicate wells.

#### 4.8.6. TEM of LysTAC1 Treated *A. baumannii*

Log phase *A. baumannii* Ab2 cells were harvested and washed twice with 20 mM Tris-HCl buffer (pH 7.0) to remove residual medium components. A portion of untreated cells was reserved as the non-EDTA, non-endolysin control group. The remaining culture was treated with 1 mM EDTA for 30 min at room temperature, followed by three washes with 20 mM Tris-HCl buffer (pH 7.0) to remove EDTA. The EDTA-treated cells were divided into two groups: one was supplemented with 100 μg/mL LysTAC1, and the other received an equal volume of 20 mM Tris-HCl buffer (pH 7.0). All groups were incubated at 37 °C for 2.5 h. After incubation, the LysTAC1-treated cells were washed three times, followed by fixation with 2.5% glutaraldehyde at 4 °C for 4 h. After 4 gentle washes to remove fixatives, cells from all groups were negatively stained with 2% phosphotungstic acid for 5 min on copper grids. Finally, morphological examination was performed using a transmission electron microscope (JEOL JEM-2100 Plus TEM, Tokyo, Japan).

### 4.9. Stability of LysTAC1

Stability assays were performed following established protocols [78,96] with minor modifications. For thermal stability, LysTAC1 was incubated at temperatures ranging from 4 °C to 100 °C (4, 25, 37, 45, 55, 65, 80, and 100 °C) for 1 h. LysTAC1 (100 μg/mL) was then added to EDTA-permeabilized *A. baumannii* Ab2 cells (1 × 10^8^ CFU/mL), incubated at 37 °C for 2.5 h, and plated with serial dilutions to assess bactericidal activity. Thermal stability was calculated relative to the bacterial killing capacity at 37 °C.

For pH stability, LysTAC1 (100 μg/mL) was pre-incubated in 20 mM Tris-HCl buffer at pH values from 3 to 11 for 1 h at room temperature. The pH was then neutralized to pH 7.0, and the pH-treated LysTAC1 was added to permeabilized *A. baumannii* Ab2 cells (1 × 10^8^ CFU/mL). Suspensions were incubated at 37 °C for 2.5 h. pH stability was determined by calculating bactericidal efficiency relative to activity at pH 7.0.

Metal ion effects were evaluated by introducing LysTAC1 (100 μg/mL) to permeabilized *A. baumannii* Ab2 cells (1 × 10^8^ CFU/mL) in the presence of various metal ions (MgCl_2_, KCl, FeSO_4_, and CaCl_2_) at concentrations of 0.00, 1.00, and 100 mM. After incubation at 37 °C for 2.5 h, bacterial viability was assessed by standard plating methods. All stability assays were conducted in technical triplicate with three independent biological replicates, and relative LysTAC1 activity was calculated using the standard formula.

Relative activity (%) = CFU reduction at test condition/CFU reduction at optimum condition × 100.

### 4.10. Statistical Analysis

All statistical analyses were conducted using GraphPad Prism software version 10.1.2, employing an unpaired *t*-test and one-way ANOVA. Significance analyses were performed with *p* values of <0.05 (*), <0.01 (**), <0.001 (***), and <0.0001 (****).

## 5. Conclusions 

We functionally characterized LysTAC1, a novel chitinase domain-containing endolysin derived from *A. baumannii* phage TAC1. LysTAC1 demonstrated stability and potent antibacterial activity against MDR-*A. baumannii* and *E. coli* in combination with membrane permeabilizers, EDTA. Its structural uniqueness highlights its potential as a promising candidate for antimicrobial development.

## Figures and Tables

**Table 1:** Bacterial strains, plasmid, and primers used in this study. CRC (colorectal cancer).

Strains, Plasmid, Primers	Description, Characteristics, and Sequence	Origin and Reference
**Strains**		
*A. baumannii*	11 clinical isolates	Sputum samples (Microbiology lab, CH, DUT)
*E. coli* RW-29 *S. aureus* *E. gallinarum* HCD 28-1	Pathogenic strain Pathogenic strain Pathogenic strain	CRC patient (Laboratory collection) (not published) ATCC 29213 Cholecystitis patient (Laboratory collection) [99]
*E. coli* BL21 (DE3)	For protein expression	Novagen, Madison, WI, USA
*E. coli* Top10	For cloning	ThermoFisher Scientific, Waltham, MA, USA
**Plasmid**		
pET-24b	*E. coli* expression vector, Kan^r^	Novagen, Madison, WI, USA
**Primers**		
*rpoB*		
Forward	5′-CTGACTTGACGCGTGA-3′	[100]
Reverse	5′-TGTTTGAACCCATGAGC-3′	
*Gluconolactonase*		
Forward	5-TTGGAGAATGCCCAACTTGG-3′	[100]
Reverse	5′-CCCGTCTTCGAGCGCAAC-3′	

## Data Availability

The original data for this work are available upon email request to the corresponding author.

## Supplementary Materials

The following supporting information can be downloaded at: https://www.mdpi.com/article/10.3390/antibiotics14100975/s1, Figure S1: PCR amplification products for molecular identification of clinical *A. baumannii* isolates using rpoB and gluconolactonase gene markers. Table S1: Antibiotic susceptibility profiles of clinical *A. baumannii* isolates.
