# Supplementary material for: In Vitro Antibacterial Efficacy of Recombinant Phage-Derived Endolysin LysTAC1 Against Carbapenem-Resistant Acinetobacter baumannii
Source: Antibiotics (Basel). 2025 Sep 26;14(10):975. doi: 10.3390/antibiotics14100975 (PMC12561043; doi:10.3390/antibiotics14100975)
Supplement: Supplementary file 1 [file antibiotics-14-00975-s001.zip › antibiotics-3799917-supplementary.pdf]

Supplementary

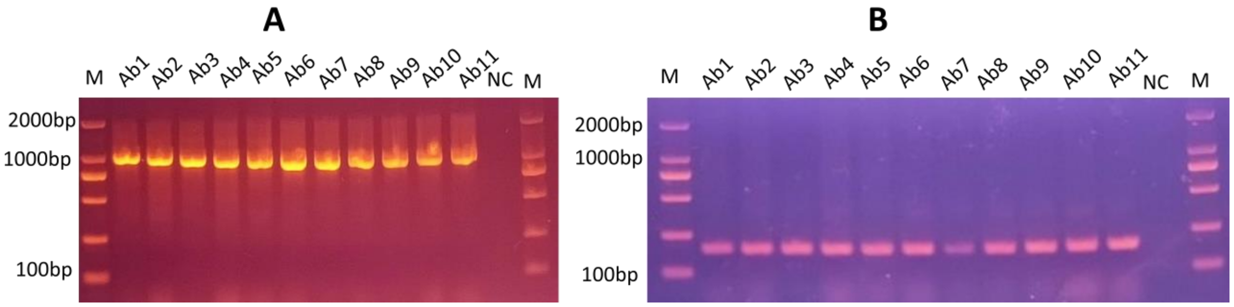

Figure S1. Molecular identification of *A. baumannii* clinical isolates. (**A**) Amplification of rpoB gene (1024bp); (**B**) amplification of gluconolactonase gene (185bp).

Isolates	PIP	CTR	IPM	AMK	GEN	TET	MI	DOX	CIP	LEV
Ab1	20mm (I)	16mm (I)	17mm (R)	17mm (S)	14mm (I)	17mm (S)	20mm (S)	21mm (S)	21mm (S)	18mm (S)
Ab2	15mm (R)	16mm (I)	15mm (R)	14mm (R)	14mm (I)	9mm (R)	14mm (I)	16mm (S)	18mm (I)	19mm (S)
Ab3	17mm (R)	14mm (I)	18mm (R)	15mm (I)	13mm (I)	8mm (R)	16mm (S)	14mm (S)	18mm (I)	13mm (R)
Ab4	10mm (R)	10mm (R)	12mm (R)	13mm (R)	10mm (R)	10mm (R)	12mm (R)	10mm (I)	8mm (R)	8mm (R)
Ab5	12mm (R)	8mm (R)	8mm (R)	12mm (R)	7mm (R)	11mm (R)	12mm (R)	8mm (R)	12mm (R)	10mm (R)
Ab6	13mm (R)	12mm (R)	15mm (R)	15mm (I)	14mm (I)	14mm (I)	14mm (I)	7mm (R)	10mm (R)	12mm (R)
Ab7	17mm (R)	13mm (R)	20mm (I)	12mm (R)	13mm (I)	14mm (I)	16mm (S)	15mm (S)	15mm (R)	15mm (I)
Ab8	12mm (R)	9mm (R)	10mm (R)	15mm (I)	13mm (I)	9mm (R)	12mm (R)	12mm (I)	11mm (R)	10mm (R)
Ab9	18mm (I)	12mm (R)	16mm (R)	16mm (I)	14mm (I)	12mm (I)	17mm (I)	20mm (I)	19mm (I)	13mm (R)
Ab10	15mm (R)	11mm (R)	12mm (R)	10mm (R)	12mm (R)	7mm (R)	8mm (R)	6mm (R)	13mm (R)	11mm (R)
Ab11	13mm (R)	6mm (R)	13mm (R)	17mm (S)	13mm (R)	9mm (R)	11mm (R)	9mm (R)	15mm (R)	9mm (R)

Table S1. Antibiotic susceptibility pattern of *A. baumannii* clinical isolates.

PIP: Piperacillin; CTR: Ceftriaxone; IPM: Imipenem: AMK: Amikacin; GEN: Gentamicin; TET: Tetracycline; MI: minocycline; DOX: Doxycycline; CIP: Ciprofloxacin; LEV:

Levofloxacin.

The interpretive criteria used for *A. baumannii* according to CLSI M100- Ed 34: piperacillin ≥21mm susceptible (S), 18-20mm intermediate (I), ≤17mm resistant (R); ceftriaxone ≥21mm (S), 14-20mm (I), ≤13mm (R); imipenem ≥22mm (S), 19-21mm (I), ≤18mm (R); amikacin ≥17 (S), 15-16mm (I), ≤14mm (R); gentamicin ≥15, 13-14mm (I), ≤12mm (R); tetracycline ≥15mm (S), 12-14mm (I), ≤11mm (R); minocycline ≥16 (S), 13-15mm (I), ≤12mm (R); doxycycline ≥13mm (S), 10-12mm (I), ≤9mm (R); ciprofloxacin ≥12mm, 16-20mm (I), ≤15mm (R), and levofloxacin ≥17mm (S), 14-16mm (I), ≤13mm (R).
